# Characterization of the Angiogenic Potential of Human Regulatory Macrophages (Mreg) after Ischemia/Reperfusion Injury In Vitro

**DOI:** 10.1155/2019/3725863

**Published:** 2019-06-25

**Authors:** Lars Hummitzsch, Karina Zitta, Rene Rusch, Jochen Cremer, Markus Steinfath, Justus Gross, Fred Fandrich, Rouven Berndt, Martin Albrecht

**Affiliations:** ^1^Department of Anesthesiology and Intensive Care Medicine, University Hospital of Schleswig-Holstein, Kiel, Germany; ^2^Department of Cardiovascular Surgery, University Hospital of Schleswig-Holstein, Kiel, Germany; ^3^Clinic for Vascular Surgery, Bad Segeberg, Germany; ^4^Department of Applied Cell Therapy, University Hospital of Schleswig-Holstein, Kiel, Germany

## Abstract

Ischemia/reperfusion- (I/R-) induced organ damage represents one of the main causes of death worldwide, and new strategies to reduce I/R injury are urgently needed. We have shown that programmable cells of monocytic origin (PCMO) respond to I/R with the release of angiogenic mediators and that transplantation of PCMO results in increased neovascularization. Human regulatory macrophages (Mreg), which are also of monocytic origin, have been successfully employed in clinical transplantation studies due to their immunomodulatory properties. Here, we investigated whether Mreg also possess angiogenic potential in vitro and could represent a treatment option for I/R-associated illnesses. Mreg were differentiated using peripheral blood monocytes from different donors (*N* = 14) by incubation with M-CSF and human AB serum and stimulation with INF-gamma. Mreg cultures were subjected to 3 h of hypoxia and 24 h of reoxygenation (resembling I/R) or the respective nonischemic control. Cellular resilience, expression of pluripotency markers, secretion of angiogenic proteins, and influence on endothelial tube formation as a surrogate marker for angiogenesis were investigated. Mreg showed resilience against I/R that did not lead to increased cell damage. Mreg express DHRS9 as well as IDO and display a moderate to low expression pattern of several pluripotency genes (e.g., NANOG, OCT-4, and SOX2). I/R resulted in an upregulation of IDO (*p* < 0.001) while C-MYC and KLF4 were downregulated (*p* < 0.001 and *p* < 0.05). Proteome profiling revealed the secretion of numerous angiogenic proteins by Mreg of which several were strongly upregulated by I/R (e.g., MIP-1alpha, 19.9-fold; GM-CSF, 19.2-fold; PTX3, 5.8-fold; IL-1*β*, 5.2-fold; and MCP-1, 4.7-fold). The angiogenic potential of supernatants from Mreg subjected to I/R remains inconclusive. While Mreg supernatants from 3 donors induced tube formation, 2 supernatants were not effective. We suggest that Mreg may prove beneficial as a cell therapy-based treatment option for I/R-associated illnesses. However, donor characteristics seem to crucially influence the effectiveness of Mreg treatment.

## 1. Introduction

Ischemic organ injuries like myocardial infarction and stroke contribute to the main causes of death in industrial countries [1]. An early restoration of blood flow to the ischemic organ by pharmacological and interventional thrombolysis, stent implantation, or bypass surgery is essential to prevent irreversible tissue damage and consequent organ failure. Although tissue ischemia already induces a proinflammatory immune response in order to clear necrotic cells and matrix debris from the infarcted area, reperfusion paradoxically amplifies the initial tissue damage, resulting in so-called ischemia/reperfusion (I/R) injury [2, 3].

To limit the I/R-induced organ damage, the development of novel treatment strategies is under intensive investigation. Besides ischemic/pharmacological preconditioning and antioxidative/immune-suppressive therapies, cell-based approaches aiming at the regeneration of functionally impaired tissues in organs affected by I/R injury are emerging treatment options [2, 3].

Stem cells are defined as cells that possess the ability of self-renewal and transdifferentiation into specialized cell types, a feature that makes stem cells an ideal tool for the treatment of degenerative or ischemic diseases. In general, possible cell candidates for stem cell therapy consist of pluripotent cells (e.g., embryonic stem cells), induced pluripotent stem cells (IPSC), and adult stem cells (e.g., hematopoietic stem cells and mesenchymal stem cells (MSC)) [4]. It is commonly accepted that the ideal cell type for a successful cell-based therapy of ischemic diseases should display the following characteristics: (i) high plasticity, (ii) ability to transdifferentiate into mature cell types in response to microenvironmental stimuli, (iii) promotion of angiogenesis and cellular survival by secretion of paracrine mediators, (iv) low immunogenicity and carcinogenicity, (v) anti-inflammatory properties, (vi) high resilience in hypoxic microenvironments, and (vii) simple generation and cultivation. Although pluripotent stem cells fulfill most of the mentioned characteristics, the main limitation of pluripotent stem cells is, besides ethical and political concerns, the associated risk of teratoma formation [5, 6]. The latter drawback does also apply to IPSC [7]. Therefore, up to now, MSC have become the most investigated and promising cell types for the treatment of ischemic diseases like myocardial infarction [4]. MSC are mesoderm-derived stromal cells that possess multidifferentiation potential, immunomodulatory properties, low carcinogenicity, and immunogenicity allowing allogenic transplantation. However, the therapeutic effects of MSC transplantation, especially in I/R-mediated organ damage, are still mostly unclear [4]. MSC only poorly transdifferentiate (e.g., into cardiomyocytes) and exhibit very low survival rates after transplantation into ischemic areas [4, 8]. Despite the low cellular resilience in hypoxic microenvironments and poor differentiation into functional cells, several studies could demonstrate positive effects after MSC transplantation, suggesting paracrine secretion of angiogenic and anti-inflammatory mediators to be responsible for the observed beneficial effects [4, 5].

Another potential cell types that might be suitable for cell-based therapy are monocyte-derived cells. These cells can be isolated in large numbers from peripheral blood and are suitable for both autologous and allogeneic therapies [9]. In general, the monocyte/macrophage axis is particularly involved in the process of neoangiogenesis and tissue renewal after I/R injury like myocardial infarction [9, 10]. It has been demonstrated that peripheral blood monocytes can increase their state of plasticity and expression of various markers of pluripotency after growth factor-induced reprogramming in vitro. The incubation of monocytes with M-CSF, IL-3, and human serum for 4-6 days results in an upregulation of pluripotency genes like C-MYC, OCT-4, and NANOG [11]. These cells are called “programmable cells of monocytic origin (PCMO)” and can be differentiated into hepatocyte-like, insulin-producing, and chondrocyte-like cells by incubation with specific cultivation media [12, 13]. Recently, we have shown that PCMO are also very resistant against hypoxic conditions and respond to I/R with the release of proangiogenic mediators inducing in vitro angiogenesis. Additionally, we demonstrated that transplantation of PCMO into chronic ischemic hind limbs of mice resulted in increased neovascularization and improved tissue oxygenation [14]. Another promising cell type that could be appropriate for cell-based therapies of I/R injury-related diseases are the so-called regulatory macrophages (Mreg). Similar to PCMO, Mreg originate from peripheral blood monocytes and can be generated by incubation with M-CSF and human serum and an additional short-term stimulation with IFN-gamma. Hutchinson and colleagues have described immunomodulatory properties of Mreg, suggesting Mreg as ideal cells for immunosuppressive therapies in solid organ transplantation [15, 16]. Several clinical studies have demonstrated that administering Mreg cell preparations to renal transplant recipients is safe and well-tolerated [15, 17]. Based on these promising initial trials, a large-scale collaborative project (ONE Study) funded by the European Community is currently investigating an array of novel manufactured cell therapy products (including Mreg: ClinicalTrials NCT02085629) regarding their potential to reduce the life-long dependency of kidney transplant patients on immunosuppressive drugs [18].

Due to their cellular characteristics, similarities with PCMO, and promising results from clinical trials, we hypothesize that Mreg cells could also be employed in the cell-based therapy of I/R injury. Therefore, in the study presented, we characterized the angiogenic and regenerative potential of in vitro cultured Mreg under normoxic and hypoxic conditions.

## 2. Material and Methods

### 2.1. Ethics

The study was authorized by the local Ethics Committee of the University Medical Center Schleswig-Holstein, Kiel, Germany (protocol numbers: D519/18 and D518/13).

### 2.2. Experimental Setting

Peripheral blood monocytes were isolated from leukapheresis products from the Department of Transfusion Medicine (University Hospital of Schleswig-Holstein, Kiel, Germany) by Ficoll-Paque PLUS (GE Healthcare, Chicago, USA) density gradient centrifugation and selective adherence to cell culture surfaces according to established protocols [19].

**Figure 1:**
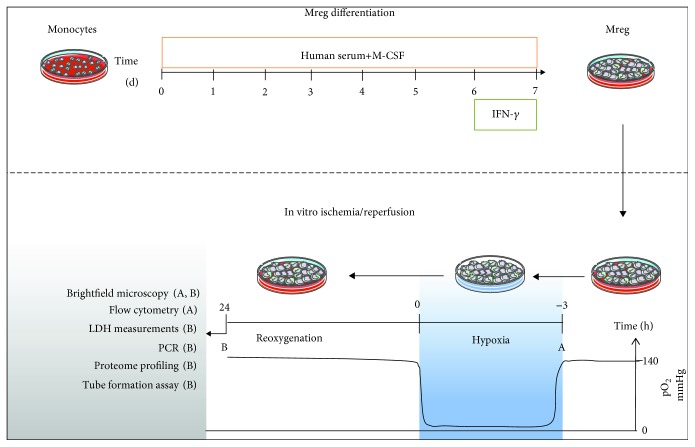
Experimental setting.

Monocytes (160,000 cells/cm^2^) were cultured in RPMI-1640 medium containing 10% human AB serum, 5 ng/ml M-CSF, 2 mM L-glutamine, 100 U/ml penicillin, and 100 *μ*g/ml streptomycin. On day 6 of culture, 25 ng/ml IFN-gamma were administered to the cultured cells. After 24 h of IFN-gamma stimulation, in vitro I/R was induced utilizing our previously described two-enzyme system with minor modifications [20]. Briefly, for induction of hypoxia, cell culture medium was exchanged by medium containing 120 U/ml catalase (Sigma-Aldrich, Schnelldorf, Germany) and 2 U/ml glucose oxidase (Sigma-Aldrich), resulting in a rapid decrease of partial pressure of oxygen (pO_2_) below 10 mmHg. Moreover, using this system, pH and glucose concentration in the culture medium decrease, resembling well the in vivo situation of I/R [21]. Hypoxia was verified by using a tissue oxygen pressure monitor (LICOX CMP Oxygen Catheter, Integra, Plainsboro, USA) and terminated by replacing the hypoxic medium with standard culture medium, resulting in an immediate reoxygenation as well as increase of pH and glucose concentration in the cultures. Control experiments were performed under normoxia by omitting the hypoxia-inducing enzymes (glucose oxidase and catalase) from the respective culture media (Figure 1).

### 2.3. LDH Cytotoxicity Assays

The colorimetric Cytotoxicity Detection Kit PLUS (Roche, Mannheim, Germany) was used for the quantification of cell damage by measuring lactate dehydrogenase (LDH) activity released from cultured cells. Preparation of samples and measurements were performed according to the manufacturer protocol. Briefly, culture media were collected 24 h after hypoxia, and samples were stored at -20°C. LDH activity of the samples was analyzed in 96-well plates at 492 nm using an ELISA reader (Tecan, Mannedorf, Switzerland) in combination with the Magellan software v1.1. The measured activities in the samples were related to the total protein content of the respective culture dishes.

### 2.4. Isolation of RNA and Reverse Transcriptase-PCR

Cells were washed twice with phosphate-buffered saline (Sigma-Aldrich) and lysed in RLT buffer (Qiagen, Hilden, Germany). RNA was isolated with the Qiagen RNeasy Minikit according to the manufacturer's protocol (Qiagen, Hilden, Germany). RNA concentrations in the samples were quantified with a spectrophotometer at 260 nm. Purity of RNA was assessed by the 260/280 nm ratio. In total, 200 ng of RNA was used to produce cDNA by a reverse transcription kit (Applied Biosystems, Carlsbad, USA) employing random hexamer primers. A 2 *μ*l sample in a final volume of 20 *μ*l was used as a template for PCR experiments, employing DNA Taq Polymerase from Solis BioDyne (Tartu, Estonia). The following primers were synthesized (metabion, Martinsried, Germany) and used to amplify specific fragments of the human transcripts: forward 5 ′-TGACCGACCCAGAGAATGTCA-3 ′ and reverse 5 ′-GCCGGGAACACCAGCATTATT-3 ′ for *DHRS9*, forward 5 ′-ATGCAGACTGTGTCTTGGCA-3 ′and reverse 5 ′-GCGCCTTTAGCAAAGTGTCC-3 ′ for *IDO*, forward 5 ′-GATTTGTGGGCCTGAAGAAAACT-3 ′ and reverse 5 ′-AAAGGCTGGGGTAGGTAGGT-3 ′ for *NANOG*, forward 5 ′-CTTGGCGGGAAAAAGAACGG-3 ′ and reverse 5 ′-TTCTCCTCCTCGTCGCAGTA-3 ′ for *C-MYC*, forward 5 ′-GGCCACACGTAGGTTCTTGA-3 ′ and reverse 5 ′-GAATACCTTCCCAAATAGAACCCC-3 ′ for *OCT-4*, forward 5 ′-GTCAGTCCCGGGGATTTGTA-3 ′ and reverse 5 ′-ATGCTCGGTCGCATTTTTGG-3 ′ for *KLF4*, forward 5 ′-TTCATCGACGAGGCTAAGCG-3 ′ and reverse 5 ′-CATCATGCTGTAGCTGCCGT-3 ′ for *SOX2*, and forward 5 ′-GTTGGTGGAGCGATTTGTCTGG-3 ′ and reverse 5 ′-AGGGCAGGGACTTAATCAACGC-3 ′ for *18sRNA*. All PCR products were separated on 2.5% agarose gels containing 0.005% Roti®-Safe GelStain (Carl Roth) and were visualized by UV-transillumination. Images were taken and densitometrically analyzed with the software ImageJ (v1.41, NIH).

### 2.5. Profiling of Angiogenesis-Related Proteins

Analysis of secreted angiogenesis-related proteins was performed using human angiogenesis proteome profiling arrays (ARY007, R&D Systems) according to the manufacturer's protocol provided with the assay kit. After culturing Mreg as described above, 600 *μ*l of cell culture supernatant was applied to the respective array membrane. Samples from Mreg supernatants from 5 different donors (D1, D2, D8, D13, and D14) were analyzed separately. Expression levels of 55 angiogenesis-associated proteins were evaluated by densitometric analyses of the arrays using the ImageJ 1.41 software (NIH). For each spot on the membrane, the optical density was measured, and the cutoff signal level was set above 10% of the respective reference spots.

### 2.6. Isolation of Human Umbilical Vein Endothelial Cells (HUVEC)

HUVEC were isolated from umbilical cords as described previously [22] and cultured in endothelial cell growth medium (ECGM) (PromoCell, Heidelberg, Germany) supplemented with 4 *μ*l/ml of endothelial cell growth supplement, 0.1 ng/ml epidermal growth factor, 1 ng/ml basic fibroblast growth factor, 90 *μ*g/ml heparin, 1 *μ*g/ml hydrocortisone (all from PromoCell), and 10% fetal bovine serum (Thermo Fisher, Schwerte, Germany). The cells were cultured in a humidified atmosphere with 5% carbon dioxide at 37°C. For further experiments, cells were detached by using Accutase (Innovative Cell Technologies, San Diego, USA) and seeded in respective cell culture plates.

### 2.7. Endothelial Tube Formation Assays

HUVEC were harvested as described above and resuspended in the respective Mreg conditioned supernatants diluted 1 : 1 with HUVEC culture media. 110 *μ*l of cell suspension containing 10,000 cells was transferred into each well of a tube formation chamber (ibidi GmbH, Munich, Germany) coated with Matrigel. Photomicrographs of the cells were taken after 7 h of cultivation in (I) cell culture media from Mreg that were subjected to I/R in vitro (I/R media, IRM), (II) cell culture media from Mreg that were subjected to normoxia in vitro (normoxic media, NM), and (III) nonconditioned control monocyte medium (control medium, CM). Angiogenic parameters (number of branches, number of pieces, number of segments, and number of junctions) were evaluated from each picture using the angiogenesis analyzer of the ImageJ software 1.41 (NIH) as described previously [23].

### 2.8. Statistical Analyses

All values are expressed as the mean ± standard error of the mean (SEM). Data were analyzed with the GraphPad Prism version 5.01 for Windows (GraphPad Software; San Diego, California, USA) and tested for normality using the Kolmogorov-Smirnov test before employing parametric tests. Statistical comparisons of two groups were performed using Student's *t*-test or the One-sample *t*-test. A *p* value < 0.05 was considered significant.

## 3. Results

### 3.1. Resilience of Mreg after I/R

**Figure 2:**
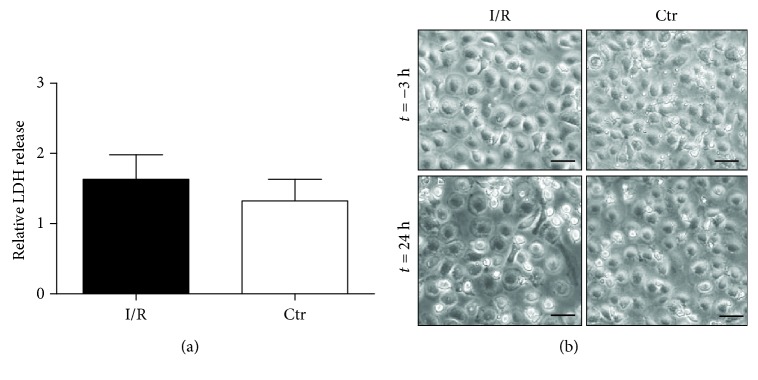
Effects of I/R on LDH release and morphology of Mreg. (a) Relative LDH release. (b) Phenotypic characterization of Mreg. Scale bars in the photomicrographs denote 40 *μ*m. I/R: ischemia/reperfusion; Ctr: control; *N* = 14.

To quantify cellular disintegration of Mreg after I/R injury, LDH activity as a marker of cell damage was quantified in cell culture media and related to the total protein content within the cultures to account for the respective numbers of Mreg. LDH activity measured within the culture supernatants did not differ between Mreg subjected to I/R in vitro and the control conditions, suggesting high resilience of Mreg after I/R injury (I/R: 1.63 ± 0.35 a.u., Ctr.: 1.33 ± 0.31 a.u.; *p* > 0.05, Figure 2(a)). Morphology of Mreg was slightly altered in the I/R group, and Mreg represented a more attached and slightly flattened and elongated phenotype (Figure 2(b)).

### 3.2. Effects of I/R on Mreg Gene Expression

**Figure 3:**
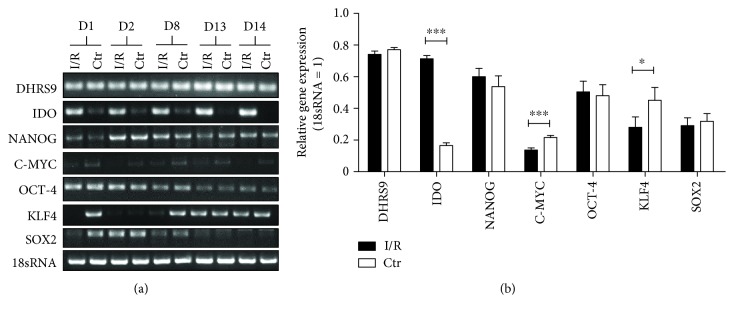
Influence of I/R on gene expression. (a) Respective PCR results from Mreg derived from 5 different donors (D1, D2, D8, D13, and D14). (b) Graphical and statistical analysis of gene expression in Mreg under control conditions and after I/R. I/R: ischemia/reperfusion; Ctr: control; ^∗^ *p* < 0.05; ^∗∗∗^ *p* < 0.001; *N* = 14.

To evaluate the effects of I/R injury on Mreg gene expression, semiquantitative analysis (relative gene expression, related to 18sRNA) of genes involved in regulation of cell plasticity (NANOG, C-MYC, OCT-4, KLF4, and SOX2), Mreg stability (DHRS9), and immunomodulatory capability (IDO) was performed. Whereas the regulatory genes NANOG (I/R: 0.60 ± 0.05, Ctr.: 0.54 ± 0.07) and OCT-4 (I/R: 0.50 ± 0.07, Ctr.: 0.48 ± 0.07) were only moderately expressed and not regulated by I/R, the expression of C-MYC (I/R: 0.14 ± 0.01, Ctr.: 0.22 ± 0.01; *p* < 0.001), KLF4 (I/R: 0.28 ± 0.07, Ctr.: 0.45 ± 0.08; *p* < 0.05), and SOX2 (I/R: 0.28 ± 0.05, Ctr.: 0.32 ± 0.05) was low under control conditions, and expression of KLF4 and C-MYC was significantly downregulated after I/R injury (Figure 3). In our experiments, the DHRS9 gene was highly expressed in Mreg after I/R and under control conditions (I/R: 0.74 ± 0.02, Ctr.: 0.77 ± 0.01). The immunosuppressive properties of Mreg are partly based on an upregulation of indoleamine 2,3-dioxygenase (IDO) activity [16]. Interestingly, our results revealed a significant upregulation of IDO gene expression after I/R (I/R: 0.71 ± 0.02, Ctr.: 0.17 ± 0.01; *p* < 0.001). Additional flow cytometry analysis confirmed the cell type-specific surface marker expression (Supplement 1).

### 3.3. Release of Angiogenic Proteins

**Figure 4:**
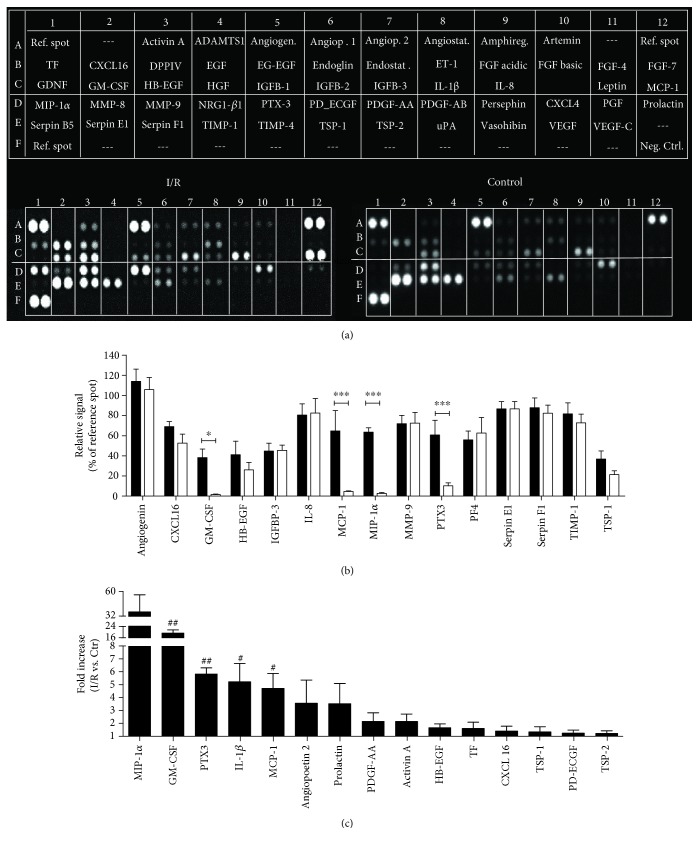
Evaluation of the effects of I/R on angiogenic protein secretion. (a) Analyzed angiogenesis-related proteins and location on the respective array membranes; the lower panel shows one representative array (out of *N* = 5). (b) Angiogenic proteins with high levels of secretion. (c) Top 15 I/R-regulated angiogenic proteins. All proteins are presented as duplicate spots on the respective array membranes. For a detailed description of the array proteins, please refer to Supplement 2. I/R: ischemia/reperfusion; Ctr: control. ^∗^ *p* < 0.05, ^∗∗∗^ *p* < 0.001, Student's *t*-test, ^#^ *p* < 0.05, ^##^ *p* < 0.01, one-sample *t*-test, *N* = 5.

Analyses of Mreg cell culture supernatants revealed a high secretory activity and release of angiogenesis-related proteins (Figure 4(a)). The most secreted proteins were (proteins that are regulated by I/R are shown in italic letters) angiogenin (I/R: 113.80 ± 12.27; Ctr.: 105.80 ± 11.94), CXCL16 (I/R: 69.07 ± 4.94; Ctr.: 52.60 ± 8.96), *GM-CSF* (I/R: 38.29 ± 8.45; Ctr.: 1.53 ± 0.45; *p* < 0.05), HB-EGF (I/R: 41.19 ± 13.36; Ctr.: 26.03 ± 7.40), IGFBP-3 (I/R: 44.70 ± 7.86; Ctr.: 45.46 ± 5.20), IL-8 (I/R: 80.54 ± 11.06; Ctr.: 82.45 ± 14.41), *MCP-1* (I/R: 64.73 ± 20.23; Ctr.: 4.61 ± 0.70; *p* < 0.001), *MIP-1α* (I/R: 63.54 ± 4.27; Ctr.: 2.71 ± 0.86; *p* < 0.001), MMP-9 (I/R: 71.91 ± 20.23; Ctr.: 72.50 ± 10.55), *PTX3* (I/R: 60.77 ± 14.48; Ctr.: 10.23 ± 2.91; *p* < 000.1), PF4 (I/R: 55.87 ± 8.07; Ctr.: 62.67 ± 15.39), Serpin E1 (I/R: 86.61 ± 7.29; Ctr.: 87.79 ± 12.00), Serpin F1 (I/R: 87.84 ± 9.67; Ctr.: 82.36 ± 8.03), TIMP-1 (I/R: 81.67 ± 10.86; Ctr.: 72.67 ± 8.71), and TSP-1 (I/R: 36.84 ± 7.80; Ctr.: 21.29 ± 3.84) (Figure 4(b)). The most I/R-regulated angiogenic proteins were MIP-1*α* (19.9-fold), GM-CSF (19.2-fold, *p* < 0.01), PTX3 (5.8-fold, *p* < 0.01), IL-1*β* (5.2-fold, *p* < 0.05), MCP-1 (4.7-fold, *p* < 0.05), angiopoietin-2 (3.6-fold), prolactin (3.5-fold), PDGF-AA (2.2-fold), activin A (2.1-fold), HB-EGF (1.7-fold), TF (1.6-fold), CXCL16 (1.4-fold), TSP-1 (1.3-fold), PD-ECGF (1.2-fold), and TSP-2 (1.2-fold) (Figure 4(c)). For a detailed description of the array proteins, please refer to Supplement 2.

### 3.4. Effect of Mreg-Conditioned Culture Media on Endothelial Tube Formation *In Vitro*

**Figure 5:**
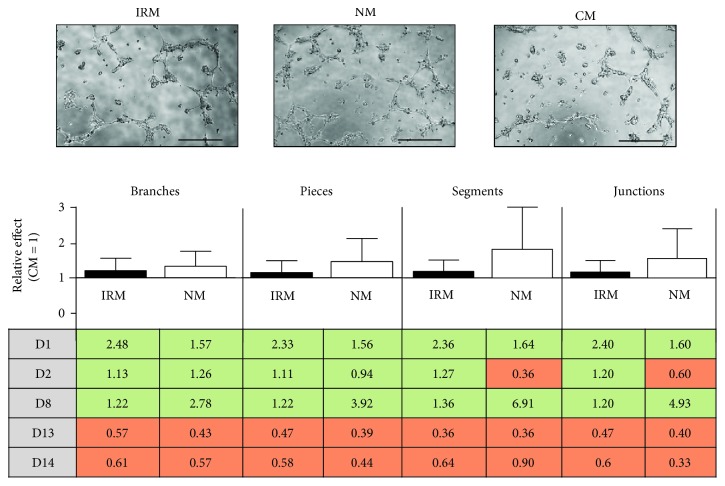
Effects of cell culture media derived from Mreg cultures subjected to I/R on endothelial tube formation. Scale bars in the photomicrographs denote 100 *μ*m. IRM: ischemia/reperfusion-conditioned media; NM: normoxia-conditioned media; CM: control media. Green color denotes an increase; orange color denotes a decrease of the respective tube formation parameter. D1, D2, D8, D13, and D14 classify the donors from which the respective Mreg were derived; *N* = 5.

Tube formation assays for the assessment of in vitro angiogenesis were performed with human umbilical vein endothelial cells (HUVEC) cultured on Matrigel-coated dishes (Figure 5). HUVEC were incubated with normoxia-conditioned media from Mreg (NM), I/R-conditioned media from Mreg (IRM), and control media (CM). There was a trend of increased formation of tube-like structures in HUVEC cultures incubated with Mreg-conditioned media (NM and IRM) without reaching statistical significance (numbers of branches, IRM/CM: 1.20 ± 0.35, NM/CM: 1.32 ± 0.42; numbers of pieces, IRM/CM: 1.14 ± 0.33, NM/CM: 1.45 ± 0.65; numbers of segments, IRM/CM: 1.32 ± 0.29, NM/CM: 2.04 ± 1.24; and numbers of junctions, IRM/CM: 1.17 ± 0.34, NM/CM: 1.57 ± 0.87; Figure 5). Interestingly, donor-based analysis of the tube formation capacity of Mreg media revealed that Mreg supernatants from 3 donors (D1, D2, and D8) induced tube formation, while 2 supernatants were not effective or even attenuated formation of tubes in HUVEC cultures (D13 and D14; table in Figure 5).

## 4. Discussion

Ischemia/reperfusion- (I/R-) induced organ damage represents one of the main causes of death worldwide and is of outstanding clinical importance. I/R injury is characterized by the production of reactive oxygen species, the release of proinflammatory mediators, and infiltration of leucocytes. An early restoration of blood flow to the ischemic organ by pharmacological or surgical intervention is essential to prevent irreversible tissue damage and consequent organ failure. Although tissue ischemia already induces a proinflammatory immune response, reperfusion paradoxically amplifies the initial tissue damage, resulting in so-called ischemia/reperfusion (I/R) injury [2, 3].

The development of novel treatment strategies to limit or prevent I/R-induced organ damage is urgently needed. Besides ischemic/pharmacological preconditioning and antioxidative/immune-suppressive therapies, cell-based therapies aiming at the regeneration of functionally impaired tissues in organs affected by I/R injury may represent promising future treatment options.

Recently, it has been hypothesized that regenerative and proangiogenic effects are induced by monocytes and their derivatives and that paracrine mechanisms rather than cellular differentiation of monocytes into vascular structures could play a central role in these mechanisms [10, 24]. Pathological processes featuring ischemic areas (i.e., myocardial infarction, critical limb ischemia, and stroke) could therefore represent ideal potential clinical targets for the use of pluripotent cells of myeloid origin. In this context, we have recently shown that in vitro differentiated programmable cells of monocytic origin (PCMO) respond to I/R with the release of angiogenic mediators and that transplantation of PCMO results in increased neovascularization of ischemic areas in a mouse model of hind limb ischemia [14].

Another monocyte-derived cell type with great promise in cell-based therapies is the human regulatory macrophages (Mreg). Mreg have already been successfully employed in clinical transplantation studies due to their immunomodulatory properties [15–18]. We hypothesized that Mreg possess angiogenic potential and may represent a possible cell therapy-based treatment option for I/R-associated illnesses.

In the study presented, we have isolated peripheral blood monocytes from different donors and differentiated these cells to Mreg by incubation with M-CSF and human AB serum and stimulation with IFN-gamma. Using our recently described enzymatic hypoxia model, Mreg cultures were subjected to 3 h of hypoxia and 24 h of reoxygenation in vitro (resembling I/R), and cellular resilience, expression of pluripotency markers, and secretion of angiogenic proteins as well as their influence on endothelial tube formation as a surrogate marker for angiogenesis were investigated.

High survival rates of transplanted cells within hostile tissues are a crucial determinant of the efficacy of cell-based therapies [8, 25]. Several studies demonstrated that due to the hypoxic and cytotoxic microenvironment present during and after ischemia, the survival rate of transplanted cells remains extensively low within the first week after transplantation [8, 26]. In our experiments, the measured LDH activity within the culture supernatants did not differ between Mreg subjected to I/R in vitro or control conditions, suggesting strong resilience of Mreg against hypoxia and possibly also I/R injury. Due to their central role in hypoxia-associated pathological processes (e.g., inflammation, arteriosclerosis, and tumor growth), macrophages possess exceptionally strong resilience within hypoxic microenvironments [26]. It seems that this unique property of macrophages is also shared by Mreg employed in our study supporting once again the potential of Mreg in cell-based therapies of ischemia-associated illnesses.

Using RT-PCR, lineage and classical markers of pluripotency were investigated in Mreg. All markers of pluripotency (NANOG, C-MYC, OCT-4, and SOX2) showed moderate or low expression after I/R and under control conditions. Likewise, KLF4 which has been reported as one of the major effectors of myeloid differentiation by various authors [27–29] also revealed low levels of expression. Riquelme et al. suggested DHRS9 as a stable marker for Mreg characterization [30]. Our results also confirm this aspect showing that DHRS9 was consistently expressed under normoxic and hypoxic conditions.

The initial phase of I/R injury is characterized by the production of reactive oxygen species, as well as a release of metabolic intermediates and cellular fragments provoking further secretion of proinflammatory mediators and infiltration of leucocytes. This primary proinflammatory immune response is then followed by an anti-inflammatory, regenerative phase characterized by tissue renewal, neoangiogenesis, and extracellular matrix production [2, 3]. There is emerging evidence that regulatory T lymphocytes (Tregs) are involved in the initiation of the reparative phase after I/R. The infiltration of Tregs into the ischemic area has been reported to be associated with several beneficial effects like suppression of proinflammatory cytokine secretion, reduction of tissue remodelling, and prevention of apoptosis [2]. Mreg exert their immunomodulatory function through their ability of transforming naïve CD4+ T-cells into IL-10-producing anti-inflammatory Tregs [16, 31]. This T-cell conversion is based on an increased indoleamine 2,3-dioxygenase (IDO) activity in Mreg [16]. Interestingly, our results revealed a significant upregulation of IDO gene expression after I/R, suggesting an increased immunomodulatory capability of Mreg especially after I/R injury.

To investigate whether Mreg are secretory active and to evaluate the effects of I/R on the release of angiogenic proteins, Mreg-conditioned culture media were characterized using angiogenesis proteome profiling arrays. Overall, most of the 55 examined proteins showed a moderate to strong secretion under control conditions as well as after I/R. However, four proteins were highly elevated in supernatants from Mreg after I/R: GM-CSF, MCP-1, MIP-1*α*, and PTX-3. Interestingly, all these factors have been strongly associated with angiogenesis and tissue recovery.

GM-CSF represents a cytokine with a wide range of biological effects, and its benefit for promoting angiogenesis in ischemic and damaged tissue has been well described [10]. Zhao et al. demonstrated that GM-CSF accelerates wound healing by promoting vascular endothelial growth factor-A (VEGF-A) expression and proliferation of endothelial cells [32]. Moreover, Ito and coworkers demonstrated that GM-CSF increases vascular collateral flow and conductance, as shown in a short-term administration of the cytokine in occlusive peripheral artery disease [33]. Finally, it has also been demonstrated that GM-CSF can play a beneficial role by inducing a phenotypic switch of inflammatory monocytes to reparative type II macrophages (M2), thereby affecting endothelial formation and neoangiogenesis in injured tissue [34].

Likewise, MCP-1 and MIP-1*α* have been identified in the pathways of angiogenesis and in response to vascular inflammation by promoting the accumulation of cells with angiogenic potential [35]. Although MIP-1*α* has frequently been associated with angiogenesis, the underlying (patho)-physiological mechanisms are still unclear [36]. Interestingly, in ischemic areas, local production of MCP-1 is enhanced, and monocytes are recruited via a MCP-1/CCR2-dependent mechanism, which is associated with a second wave of monocyte migration [37]. It may be hypothesized that the migrating monocytes are quickly converted to anti-inflammatory macrophages (M2) at the site of injury where they participate in neoangiogenesis [37].

In contrast, the influence of PTX-3 on angiogenesis has been controversially discussed until now. Accordingly, Salio et al. reported that lack of PTX-3 reduced the number of capillaries in reperfused cardiac tissue and resulted in a detrimental outcome in a study of cardiac ischemia [38]. Likewise, PTX-3 has also been described to be a factor that promotes neurogenesis and angiogenesis in neuronal tissue [39].

In accordance with the results from the angiogenesis proteome profiling arrays, tube formation assays demonstrated that supernatants from Mreg positively influence in vitro angiogenesis. There was a trend of increased formation of tube-like structures in HUVEC cultures that were incubated with normoxia-conditioned and I/R-conditioned media from Mreg. However, statistical significance was not reached, and there was also no clear difference between the effects of normoxia-conditioned and I/R-conditioned Mreg media on tube formation. Donor-based analysis of the tube formation capacity of Mreg media revealed that Mreg supernatants from 3 donors (D1, D2, and D8) induced tube formation, while 2 supernatants were not effective or even attenuated formation of tubes in HUVEC cultures (D13 and D14). It could be conceivable that variables like gender, age, and actual inflammatory state of the respective donor may influence the properties of the resulting Mreg population and therefore the outcome of the tube formation assays.

## 5. Conclusion

Our results suggest that Mreg may prove beneficial as a cell therapy-based treatment option for I/R-associated illnesses. However, donor characteristics seem to crucially influence the effectiveness of Mreg treatment, and further studies employing Mreg for cell-based therapies should focus on the selection of suitable monocyte donors as well as a detailed characterization of the resulting Mreg populations.

## Data Availability

The data used to support the findings of this study are available from the corresponding author upon request.

## Supplementary Materials

Supplementary 1 Supplement 1. Flow cytometric analysis of CD cell surface markers of Mreg. Positive cells are located above the horizontal line.

Supplementary 2 Supplement 2. Detailed description of proteins analyzed by angiogenesis arrays.
