# Supplementary figures and images for: Characterization of the Angiogenic Potential of Human Regulatory Macrophages (Mreg) after Ischemia/Reperfusion Injury In Vitro
Source: Stem Cells Int. 2019 Jun 25;2019:3725863. doi: 10.1155/2019/3725863 (PMC6614961; doi:10.1155/2019/3725863)

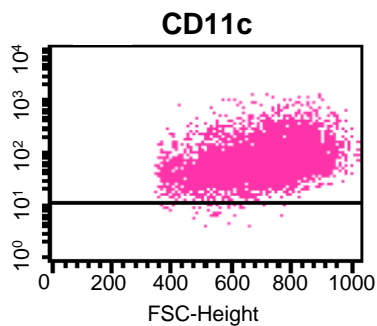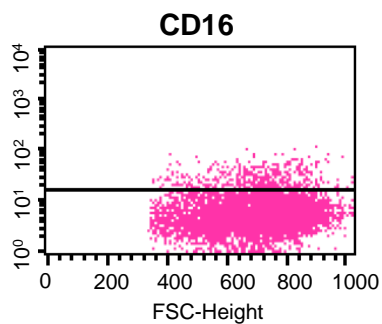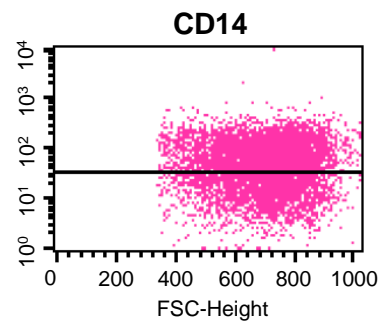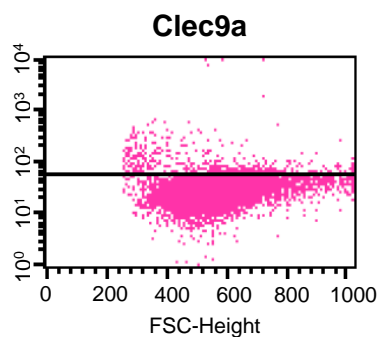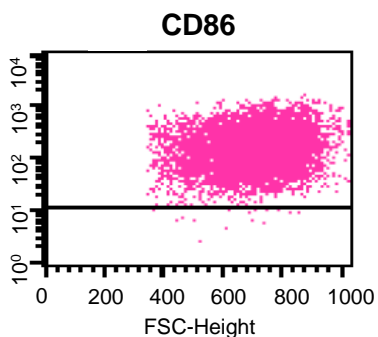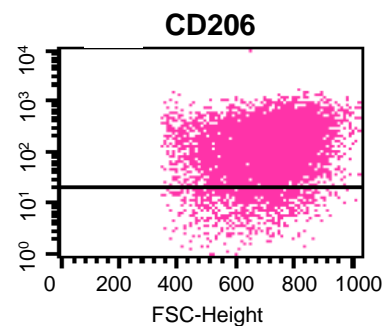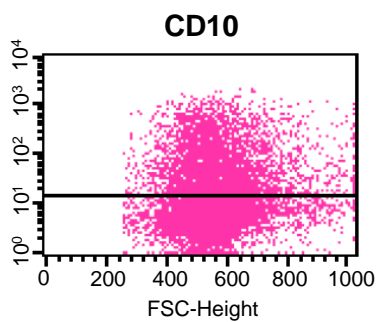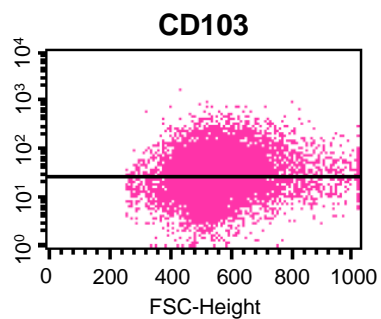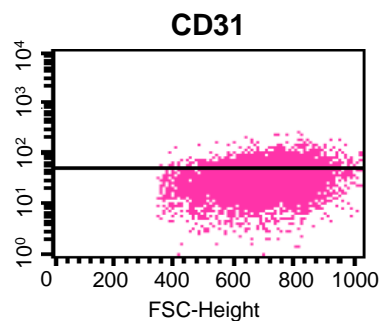

Supplement: Supplementary 1 — Supplement 1. Flow cytometric analysis of CD cell surface markers of Mreg. Positive cells are located above the horizontal line. [file 3725863.f1.pdf]
