# Supplementary material for: Characterization of the Angiogenic Potential of Human Regulatory Macrophages (Mreg) after Ischemia/Reperfusion Injury In Vitro
Source: Stem Cells Int. 2019 Jun 25;2019:3725863. doi: 10.1155/2019/3725863 (PMC6614961; doi:10.1155/2019/3725863)
Supplement: Supplementary 2 — Supplement 2. Detailed description of proteins analyzed by angiogenesis arrays. [file 3725863.f2.pdf]

Coordinate	Abbreviation	Name (alternative nomenclature)
**A1; A12; F1**	Ref. spot	Reference spot
**A3**	AktivinA	AktivinA
**A4**	ADAMTS1	Angiogenin
**A5**	Angiogenin	Angiogenin
**A6**	Angiop. 1	Angiopoetin 1
**A7**	Angiop. 2	Angiopoetin 2
**A8**	Angiostatin	Angiostatin
**A9**	Amphireg.	Amphiregulin
**A10**	Artemin	Artemin
**B1**	TF	Tissue factor (Coagulation Factor III)
**B2**	CXCL-16	C-X-C motif chemokine ligand 16
**B3**	DPPIV	Dipeptidyl-peptidase IV
**B4**	EGF	Epidermal growth factor
**B5**	EG-VEGF	Endocrine gland derived vascular endothelial growth factor
**B6**	Endoglin	Endoglin (Cluster of differentiation 105; CD105)
**B7**	Endostatin	Endostatin
**B8**	ET-1	Endothelin-1
**B9**	FGF acidic	Fibroblast growth factor acidic
**B10**	FGF basic	Fibroblast growth factor basic (FGF-2)
**B11**	FGF-4	Fibroblast growth factor-4
**B12**	FGF-7	Fibroblast growth factor-7
**C1**	GDNF	Glial cell-derived neurotrophic factor
**C2**	GM-CSF	Granulocyte-macrophage-colony stimulating factor
**C3**	HB-EGF	Heparin-binding epidermal growth factor
**C4**	HGF	Hepatocyte growth factor (Scatter factor)
**C5**	IGFBP-1	Insulin-like growth factor-binding protein 1
**C6**	IGFBP-2	Insulin-like growth factor-binding protein 2
**C7**	IGFBP-3	Insulin-like growth factor-binding protein 3
**C8**	IL-1β	Interleukin-1β
**C9**	IL-8	Interleukin-8 (C-X-C motif chemokine ligand 8; CXCL8)
**C10**	TGF-β1	Transforming growth factor-β1
**C11**	Leptin	Leptin
**C12**	MCP-1	Monocyte chemoattractant protein 1(CC-chemokine ligand 2; CCL2)
**D1**	MIP-1α	Macrophage inflammatory protein-1α (CC-chemokine ligand 3; CCL3)
**D2**	MMP-8	Matrix metalloproteinase-8
**D3**	MMP-9	Matrix metalloproteinase-9
**D4**	NRG1-β1	Neuregulin1-β1
**D5**	Pentraxin3	Pentraxin3
**D6**	PD-ECGF	Platelet-derived endothelial cell growth factor
**D7**	PDGF-AA	Platelet-derived growth factor AA
**D8**	PDGF-AB	Platelet-derived growth factor AB
**D9**	Persephin	Persephin
**D10**	CXCL4	C-X-C motif chemokine ligand 4
**D11**	PGF	Placental growth factor
**D12**	Prolactin	Prolactin
**E1**	Serpin B1	Serpin B1 (Leukocyte elastase inhibitor; LEI)
**E2**	Serpin E1	Serpin E1 (Plasminogen activator inhibitor-1 ; PAI-1)
**E3**	Serpin F1	Serpin F1 (Pigment epithelium-derived factor; PEDF)
**E4**	TIMP-1	Tissue inhibitor of metalloproteinases-1
**E5**	TIMP-4	Tissue inhibitor of metalloproteinases-4
**E6**	TSP-1	Thrombospondin-1
**E7**	TSP-2	Thrombospondin-2
**E8**	uPA	Urokinase-type plasminogen activator
**E9**	Vasohibin	Vasohibin
**E10**	VEGF	Vascular endothelial growth factor
**E11**	VEGF-C	Vascular endothelial growth factor-C
**F12**	Neg. ctr.	Negative controls
